# Pre-Cambrian roots of novel Antarctic cryptoendolithic bacterial lineages

**DOI:** 10.1186/s40168-021-01021-0

**Published:** 2021-03-19

**Authors:** Davide Albanese, Claudia Coleine, Omar Rota-Stabelli, Silvano Onofri, Susannah G. Tringe, Jason E. Stajich, Laura Selbmann, Claudio Donati

**Affiliations:** 1 Research and Innovation Centre, Fondazione Edmund Mach, Via E. Mach 1, 38098 San Michele all’Adige, Italy; 2 Department of Ecological and Biological Sciences, University of Tuscia, Largo dell’Università, 01100 Viterbo, Italy; 3 Department of Energy Joint Genome Institute, One Cyclotron Road, Berkeley, CA 94720 USA; 4 Department of Microbiology and Plant Pathology and Institute of Integrative Genome Biology, University of California, Watkins Drive 3401, Riverside, Riverside, CA 92507 USA; 5 Mycological Section, Italian Antarctic National Museum (MNA), Via al Porto Antico, 16128 Genoa, Italy

**Keywords:** Antarctica, Extremophiles, Cryptoendolithic communities, Bacteria, Evolution, Adaptation, Metagenomics, MAG, Functionality

## Abstract

**Background:**

Cryptoendolithic communities are microbial ecosystems dwelling inside porous rocks that are able to persist at the edge of the biological potential for life in the ice-free areas of the Antarctic desert. These regions include the McMurdo Dry Valleys, often accounted as the closest terrestrial counterpart of the Martian environment and thought to be devoid of life until the discovery of these cryptic life-forms. Despite their interest as a model for the early colonization by living organisms of terrestrial ecosystems and for adaptation to extreme conditions of stress, little is known about the evolution, diversity, and genetic makeup of bacterial species that reside in these environments. Using the Illumina Novaseq platform, we generated the first metagenomes from rocks collected in Continental Antarctica over a distance of about 350 km along an altitudinal transect from 834 up to 3100 m above sea level (a.s.l.).

**Results:**

A total of 497 draft bacterial genome sequences were assembled and clustered into 269 candidate species that lack a representative genome in public databases. Actinobacteria represent the most abundant phylum, followed by Chloroflexi and Proteobacteria. The “Candidatus *Jiangella antarctica*” has been recorded across all samples, suggesting a high adaptation and specialization of this species to the harshest Antarctic desert environment.

The majority of these new species belong to monophyletic bacterial clades that diverged from related taxa in a range from 1.2 billion to 410 Ma and are functionally distinct from known related taxa.

**Conclusions:**

Our findings significantly increase the repertoire of genomic data for several taxa and, to date, represent the first example of bacterial genomes recovered from endolithic communities. Their ancient origin seems to not be related to the geological history of the continent, rather they may represent evolutionary remnants of pristine clades that evolved across the Tonian glaciation. These unique genomic resources will underpin future studies on the structure, evolution, and function of these ecosystems at the edge of life.

Video abstract

**Supplementary Information:**

The online version contains supplementary material available at 10.1186/s40168-021-01021-0.

## Background

Rocks represent the earliest terrestrial niche for life on Earth when microbes were the only form of life [1, 2]. Porous rocks, in particular, remain the ultimate refuge for life in extreme environments as in the ice-free areas of Antarctica, where complex life-forms became extinct about 60-30 Ma, when the continent reached the South Pole and the Antarctic Circumpolar Current was established. The McMurdo Dry Valleys, covering a surface of approximately 4800 km^2^ in Continental Antarctica, are among the most extreme regions on Earth with only minimal resources suitable for supporting life [3, 4]. Specifically, in these desert areas, where soils have been eroded by glaciers and strong winds, life is confined to the endolithic niche that provides microorganisms with thermal buffering, physical stability, protection from abiotic stresses, and access to mineral nutrients, rock moisture and growth surfaces [5, 6]. Indeed, the endolithic environment is a ubiquitous habitat for microorganisms in dryland systems [7], but in the harshest terrestrial climates, characterized by extreme environmental conditions typically incompatible with an active life, it is often the primary or even exclusive refuge for life [8].

Endolithic microbial communities are self-sustaining ecosystems relying on the phototrophic activity of microalgae and cyanobacteria as primary producers which support a diversity of consumers including fungi, bacteria, and archaea [9–11]. In the Antarctic desert areas, the Lichen-Dominated Communities (LDC) are the most complex and successful [5]. Recently, next-generation sequencing studies have brought new insights into their composition, showing that lichens in the Lecanoromycetes and free-living fungi in the Dothideomycetes (Ascomycota) are the dominant eukaryotes, while Actinobacteria and Proteobacteria are the most abundant prokaryotes [12, 13]. Due to their ubiquity in deserts and low taxonomic complexity and biodiversity [14], endoliths are important study systems to understand evolutionary processes in the early history of life, to model how life evolves during the progression of desertification and when the extreme aridity approaches the limits of life, providing also a model for searching life elsewhere in the solar system. However, the understanding of the microbial biodiversity in these communities is limited and our comprehension about their physiology, evolution, and stress responses is still at its infancy [15].

**Fig. 1:**
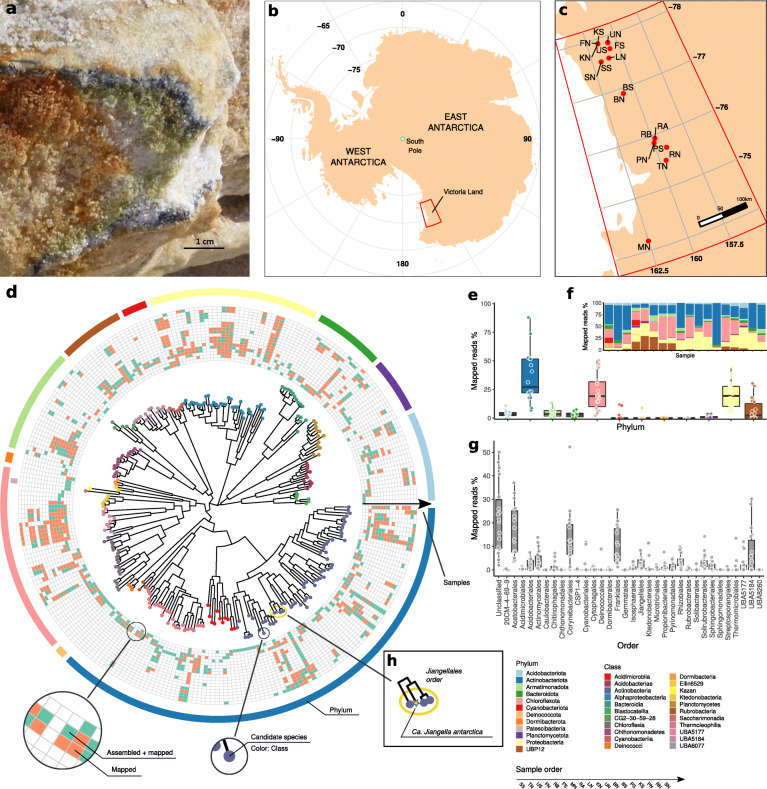
**a** Cryptoendolithic lichen-dominated community colonizing sandstone at Linnaeus Terrace, McMurdo Dry Valleys, Southern Victoria Land, Continental Antarctica. **b** Map of Antarctica. The area encircled by the red lines represents Victoria Land. **c** Map of the study area showing the location of the sampling sites. Sampled sites are listed in Supplementary Table 4. **d** Phylogenetic tree built from the GTDB-Tk multiple sequence alignment (MSA) of the 269 CBS representatives. Tip points are colored according to the GTDB taxonomic classification at the class level. Phylum-level classification is indicated by the colors in the outer circle. The 18 inner circles represent distribution of each CBS in the samples. Presence inferred by the assembly is indicated in green, while presence inferred only from the alignment of the read to the CBS representative genome is indicated in orange. **e** Percentage of reads that could be mapped to the CBS representatives, grouped by Phylum. **f** Per sample percentage of the reads that could be mapped to the CBS representatives, grouped by Phylum. **g** Same as **e**, at the order taxonomic level. **h** Jiangellales CBS, including the Candidatus *Jiangella antarctica* (yellow star)

In this study, we performed metagenomic sequencing of eighteen LDC-colonized rock samples collected in Antarctic ice-free areas (Fig. 1a) distributed over a distance of 350 km (Fig. 1b,c) to provide the first survey of the genomic repertoire of bacteria from Antarctic endolithic ecosystems [16]. The metagenomic assemblies generated more than 10 million contigs which were binned into 497 novel bacterial genomes and classified as 269 previously unknown species-level clusters, substantially expanding the sampled genomic diversity within 33 bacterial orders.

While interest is increased in the ecological roles, diversity, conservation, and biotechnological potential of the Antarctic endolithic microbiota, the evolutionary origins are still unexplored. We used molecular clock analysis to characterize the time scale over which these taxa have differentiated from known related species and to test two fundamental hypotheses on the processes that led to the establishment of these communities: (i) Antarctic endolithic bacteria may have evolved from generalist species in response to the climatic changes occurring when Antarctica reached the South Pole and (ii) these taxa could have been selected from pre-existing extremo-tolerant species. Our results give clear evidence of ancient divergence of most Antarctic bacterial clades which date back up to 1.2 billion Ma. This evidence clearly supports the second hypothesis and excludes that they are the result of a recent evolution of genetic traits in response to environmental conditions.

## Methods

### Sampling area

Victoria Land is a region of Continental Antarctica which fronts the western side of the Ross Sea and the Ross Ice Shelf; this land is positioned between the Polar Plateau and the coast and is exposed to a wide spectrum of climatic extremes, including low and fluctuating temperature, scarce precipitation regimes, and strong winds; the region covers a latitudinal gradient of 8° from Darwin Glacier (78° 00′) to Cape Adare (70° 30′ S) [17]. Ice-free areas dominate the landscape of Southern Victoria Land and the high-altitude locations of Northern Victoria Land, while low-elevation coastal soils of Northern Victoria Land receive considerable marine and biological influence (e.g., sea birds).

Sandstone rocks were collected by L. Selbmann in Victoria Land along a latitudinal transect ranging from 74° 10′ 44.0′′ S 162° 30′ 53.0′′ E (Mt. New Zealand, Northern Victoria Land) to 77° 52′ 28.6′′ S 160° 44′ 22.6′′ E (University Valley, Southern Victoria Land) during the XXXI Italian Antarctic Expedition (Dec. 2015–Jan. 2016). Samples were collected at different conditions namely sun exposure and an altitudinal transect, from 834 to 3100 m a.s.l. to provide a comprehensive overview of endolithic diversity (Fig. 1a–c). Rocks were excised using a geologic hammer and sterile chisel, and rock samples, preserved in sterile plastic bags, transported, and stored at −20 °C in the Culture Collection of Antarctic fungi of the Mycological Section of the Italian Antarctic National Museum (MNA-FCC), until downstream analysis.

### DNA extraction, library preparation, and sequencing

DNA was extracted from three samples for each site and then pooled. Metagenomic DNA was extracted from 1 g of crushed rocks using a MoBio Powersoil kit (MOBIO Laboratories, Carlsbad, CA, USA). The quality of the DNA extracted was determined by electrophoresis using a 1.5% agarose gel and with a spectrophotometer (VWR International) and quantified using the Qubit dsDNA HS Assay Kit (Life Technologies, USA).

Shotgun metagenomic libraries were prepared and sequenced at the DOE Joint Genome Institute (JGI) as a part of a Community Science Project (PI: Laura Selbmann; co-PI: Jason E. Stajich) at JGI [16]. Paired-end sequencing libraries were constructed and sequenced as 2×150 bp using the Illumina NovaSeq platform (Illumina Inc, San Diego, CA).

### Sequencing reads preparation and assembly

BBDuk (http://sourceforge.net/projects/bbmap/) v38.25 was used to remove contaminants, trim adapters, and low-quality sequences. The procedure removed reads that contained 4 or more “*N*” bases, had an average quality score across the read less than 3, or had a minimum length ≤ 51 bp or 33% of the full read length. Filtered and trimmed paired-end reads were error corrected using BFC [18] r181 with parameters -1 -s 10g -k 21 -t 10 and orphan reads were removed. Samples were assembled individually with SPAdes [19] 3.12.0 using the parameters -m 2000 -o spades3 --only-assembler -k 33,55,77,99,127 --meta -t 32.

### Binning

Metagenomic contigs were binned into candidate metagenome-assembled genomes (MAGs) using MetaBAT2 [20] (Metagenome Binning based on Abundance and Tetranucleotide frequency) v2.12.1. Briefly, high-quality reads were mapped on assembled contigs using Bowtie2 [21] v2.3.4.3. Samtools [22] v1.3.1 (htslib v1.3.2) was used to create and sort the BAM files (.bam). The depth of coverage was estimated by applying the jgi_summarize_bam_contig_depths tool. Contigs sequences and the depth of coverage estimates were used by MetaBAT2 to recover the candidate MAGs.

### Quality assessment and dereplication

Completeness and contamination estimates of bacterial and archaeal MAGs were obtained by CheckM [23]. According to recent guidelines [24], MAGs were classified into “high-quality draft” (HQ) with >90% completeness and <5% contamination and “medium-quality draft” (MQ) with completeness estimates of ≥50% and less than 10% contamination. Candidate bacterial species (CBS) were identified by clustering HQ and MQ MAGs at species level [25] (>95% Average Nucleotide Identity - ANI) using dRep [26] v2.0.0. For each CBS, the MAG with the highest quality score was chosen as representative.

### Taxonomic classification

MQ and HQ MAGs were taxonomically classified using the genome taxonomy database toolkit [27, 28] (GTDB-Tk) v0.1.6 and the GTDB release 86, following the recently proposed nomenclature of prokaryotes [29, 30]. GTDB-Tk classifies a query genome combining its placement in the GTDB reference tree (release 86 includes a total of 21,263 genomes in the tree), its RED, and its ANI to reference genomes. Approximately-maximum-likelihood phylogenetic tree from the GTDB protein alignments of the 269 CBS representatives (Fig. 1) and of the orders acetobacterales (Fig. 4) and Frankiales (Fig. S1) were inferred using FastTree [31] v2.1.10 (WAG+CAT model, options -wag -gamma) and rooted at midpoint.

### Percentage of mapping reads and CBS detection

For each metagenomic sample, high-quality reads were aligned against each CBS representative using Bowtie2 [21] v2.3.4.3 using the parameter --no-unal. Samtools v1.3.1 (htslib 1.3.2) was used to create and index the BAM files (.bam). The depth of coverage, the breadth B_n_ (i.e., the fraction of bases of the CBS representative genome that are covered with depth *n*), and the number of mapped reads were calculated on the BAM file using pysam (https://github.com/pysam-developers/pysam) v0.15.2 and Python v3.5.3. The fraction of reads mapping on a CBS representative was computed as the number of successfully aligned reads normalized by the total number of reads aligning the entire set of the CBS representatives. Regions with no coverage were identified using BEDtools [32] v2.26.0 with the options -bga -split. Variant calling was performed with samtools mpileup and bcftools call [33] (v1.3.1, options --ploidy 1 -mv). Tabix [34] v1.3.2 was used to index the output VCF file. The consensus sequence was generated using the command bcftools consensus masking the zero coverage regions previously identified. The ANI between the consensus sequence and the CBS representative (ANI_CBS_) was estimated using fastANI v1.1. Finally, a CBS was tagged as present in a sample if the breadth of coverage (at depth 2) B_2_ was ≥ 0.5 and ANI_CBS_ ≥ 95%. We detected a total of 1094 CBS distributed within the 18 metagenomic samples (see Fig. 1d, S2).

Mash Screen [35] (Mash v. 2.1) was used to validate the presence of CBS in the Antarctic samples. Briefly, we sketched all the CBS representative genomes using a sketch size of 10,000 (replacing the default value of 1000) in order to have a superior representation of the sequences [36]; after that, the metagenomes were independently screened for containment of the CBS using the command mash screen. Given a metagenome, Mash Screen reported the containment score for each CBS (i.e., the estimate of the similarity of the CBS representative to a sequence contained within the metagenome) as a proxy for the average nucleotide identity, its *p* value, and the CBS median-multiplicity as a proxy for the genome coverage. We found that 1009 out of 1094 (92.2%) detected CBS have been confirmed (containment score >0.95, *p*<1.47×10^–21^, see Fig. S5, S6, and Supplementary Table 1). The remaining 85 discoveries have containment scores >0.91, and most of them (75) have a breadth of coverage B_2_ between 0.5 and 0.7, which is compatible with the fact that Mash Screen tends to underestimate the identity when the query genome may not be fully represented by the sequencing reads [35].

### Divergence estimates

Divergence times were independently estimated on orders containing at least 4 CBS, for a total of 19 analyzed orders. For each order, we built a protein MSA using the 120 GTDB bacterial marker genes including (i) 32 reference sequences from outgroups outside the order, (ii) the GTDB representatives, (iii) the MQ and HQ Antarctic MAGs, and (iv) a set of outgroup in order to reconstruct the first radiation within bacteria as in [37] and using it as a calibration point. The 19 datasets were calibrated with this same prior. We calibrated the crown (divergence) of bacteria using a prior on the root of 3453 million years ago (Ma) and a standard deviation of 60 Ma (values kindly provided by Davide Pisani) and corresponding to the posterior estimate for the crown of the bacteria [37]. Since our taxon sampling replicates the taxon sampling in [37], we could safely apply the previous estimate for the crown of the bacteria to our root (which coincides with the crown of bacteria, as we did not use archaea or eukaryotes outgroups). Markov chain Monte Carlo (MCMC) analyses were performed using BEAST [38] v1.10 for 100 million generations sampling every 1000 generations. Convergence was assessed by using the Effective Sample Sizes (ESS) estimated by Tracer [39] v1.7.1 on posteriors and log-likelihood. In order to maximize the ESS statistics, a burn-in ranging from 50 to 80% of the simulation was used. For computational reasons, we performed model selection using only one dataset (Acidobacteriales) as representative. We compared a relaxed clock (log-normal) versus the strict clock, and a coalescence (constant) versus a speciation (birth-death) demographic model. The most fitting combination of priors (relaxed clock plus coalescence) was found using path sampling and AICM. Amino-acid substitutions were modeled using the LG matrix with amino acid frequencies inferred from the data; among-site rate variation was modeled using a gamma distribution with four discrete categories. All Bayesian posterior annotated Maximum Clade Credibility Trees are reported in Supplementary Data. For each order, the mean age (plus the 95% high posterior densities heights) for the first split of a uniquely Antarctic group (green node) from the known reference sequence from that particular order was plotted. In the case of more than one monophyletic Antarctic group, the age of the second oldest Antarctic group (orange node) was also shown.

### Functional annotation

Functional annotation was performed only on HQ CBS representatives of orders containing at least 4 CBS (for a total of 19 orders analyzed). In order to avoid systematic effects due to different annotation methods, both HQ MAGs and GTDB representative genomes (for a total of 3942 genomes) were processed as follows: (i) 16,292,642 translated coding DNA sequences (CDS) were predicted using Prokka [40] v1.13.4 which wraps the software Prodigal [41] and (ii) the CDS were functionally annotated using EggNOG-mapper [42] (option --database bact) and the eggNOG Orthologous Groups (OGs) database [43] v4.5.1. The EggNOG database integrates functional annotations collected from several sources, including KEGG functional orthologs [44], COG categories [45], and Gene Ontology (GO) terms.

In order to avoid annotation biases which are intrinsic to reference-based methods, we also clustered the CDS using MMseqs2 release 11-a29379e [46] (parameter --min-seq-id 0.60) generating 3,836,924 protein clusters. The cluster profiles were analyzed using the t-SNE dimensionality reduction (see the “Statistical analysis” section).

### Statistical analysis

Downstream analysis was performed using the R environment (https://www.R-project.org/) v3.6.1. T-SNE dimensionality reduction (Jaccard distance) on KO and 60% identity cluster profiles was carried out using the R package “tsne” (https://CRAN.R-project.org/package=tsne) v0.1-3 and the PCoA (Principal Coordinate Analysis) using the function “pcoa()” (default parameters) available in the R library “ape” v5.3. Fisher’s exact tests were conducted using the function “fisher.test()” (default parameters) available in the R package “stats” v3.6.1.

## Results

### Metagenomic assembly identifies novel bacterial species and broadly expands the tree of life

Using shotgun sequencing, we produced more than 10 million contigs that were binned into a total of 1660 metagenome-assembled genomes (MAGs), among which 497 were identified as bacterial and none as archaeal. The bacterial MAGs were partitioned into 263 high quality (HQ) and 234 medium quality (MQ) according to their estimated completeness and contamination (see the “Methods” section). Assembly, completeness and contamination statistics and the taxonomic classification of the 497 bacterial MAGs are given in Supplementary Table 2. Species-level (95% ANI cutoff, see the “Methods” section) dereplication of the MAGs produced a set of 269 clusters—or candidate bacterial species (CBS)—each represented by the MAG of highest quality. The CBS were taxonomically classified using GTDB-Tk [28] (see the “Methods” section). While all CBS could be assigned to a known phylum or class, none could be classified into existing species (Table 1). The most common phylum, both in terms of number and abundance of CBS (estimated by the fraction of mapped reads, Fig. 1e, f, Supplementary Table 3), was Actinobacteria with 101 CBS (median percentage of mapped reads 27.2%, IQR 29.5%), followed by Chloroflexi and Proteobacteria. The newly assembled MAGs increase by more than 50% the number of representative species in the Genome Taxonomy Database [27] (GTDB) for Jiangellales, Frankiales, Thermomicrobiales, Isosphaerales, Solirubrobacterales, and for the order-level UBA5184 UBA lineage [47] (Supplementary Table 4, Fig. 1g).

**Table 1:** Number of identified taxa and classified CBS for each taxonomic rank. While 100% of the CBS could be assigned to a known phylum, only 81% were classified at the genus level and none at the species level

Taxonomic rank	# of taxa	# of classified CBS (%)
Phylum	12	269 (100%)
Class	22	269 (100%)
Order	33	226 (84%)
Family	43	212 (82%)
Genus	28	81 (30%)
Species	0	0 (0%)

### Distribution of CBS among Antarctic cryptoendolithic communities

We investigated the distribution of CBS across the wide range of sampled environmental conditions (see Supplementary Table 5). Since CBS could be assembled only in samples where they had a relatively high abundance, we complemented the assembly by direct read mapping on assembled MAGs to assess presence in a given sample. Specifically, we considered a species present in a sample either (i) if an assembled genome assigned to the CBS was recovered from that sample or (ii) if the breadth of coverage of the mapped reads on the CBS representative was ≥ 50% and the ANI between the consensus sequence and the CBS was ≥ 0.95. The results of this procedure were in good agreement with the prediction of the Mash Screen algorithm [35] (see the “Methods” section and Supplementary Table 1). We identified a set of 10 CBS that were present in at least 75% (14/18) of the samples (Fig. 1d, S4, Supplementary Table 6), despite the known low sensitivity of shotgun metagenomics for the characterization of biodiversity in environmental samples [48]. This set defined a “core” of conserved species that were taxonomically classified in two phyla (Actinobacteria and Proteobacteria) and two classes, i.e., Actinobacteria and Alphaproteobacteria (Fig. S4). A member of the order Jiangellales (Actinobacteria), that herein we named “Candidatus *Jiangella antarctica*,” was present across all samples (average percentage of mapped reads 1.92%, SD 1.93%, estimated median depth of coverage from 2 to 190), Mash Screen containment *p* value <1.47×10^–21^, (Fig. 1d,h, S8, Supplementary Table 1). Extracting and classifying the nearly full-length 16S from the Ca. *Jiangella antarctica* (1,513 bp), we did not found any significant match both in the Ribosomal Database Project [49] (RDP, “unclassified Actinomycetales”) and SILVA [50] (identity of the best hit 92.09%), confirming that this species has not been previously reported. We also detected three less ubiquitous species that were related to the Antarctic *Jiangella* (Fig. 1h, Supplementary Table 6). Moreover, we found that, while all samples host at least one representative of the class Chloroflexia, three samples (SS, TN, US) host the majority of CBS from this class (Fig. 1d).

In the overall, we observed a large degree of variability among samples which appeared to host diverse bacterial assemblages. However, the majority of the CBS were detected only in a small fraction of the samples (Fig. 1d, e S4).

### Antarctic bacteria cluster in ancient monophyletic groups that evolved long before Antarctica separated from Gondwanaland

**Fig. 2:**
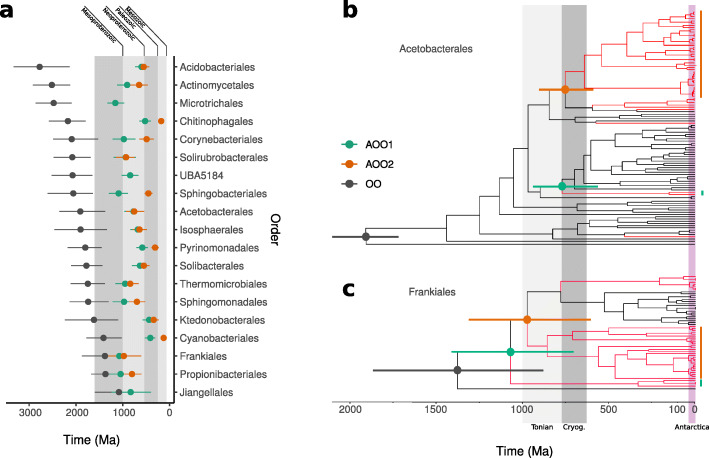
The neoproterozoic age of Antarctic cryptoendolithic clades. **a** Summary of Bayesian divergence estimates indicate that most of the oldest lineages of Antarctic cryptoendoliths originated during the Neoproterozoic (1000-541 Ma). For each of the orders we show the origin of the order (OO: the split of the order from the closest order), the origin of the oldest uniquely Antarctic clade (AOO1: the split of the Antarctic calde from a non-Antarctic lineage of the same order), and, where present, the origin of the second oldest antarctic clade (AOO2) in each order. **b** Bayesian divergence tree of the Acetobacterales and **c** of the Frankiales with the Antarctic clades highlighted in red. Time-trees for all other orders are in Supplementary Data. The shaded grey areas in **a** identify geological eras, while those in **b** and **c** identify the Tonian and Cryogenian periods of the Neoproterozoic era; pink area in **b** and **c** identify a period compatible with glacial Antarctica. Bars at nodes are uncertainties expressed as 95% of high posterior densities

For each bacterial order with at least 4 CBS (for a total of 19 orders, 377 MAGs, and 200 CBS), we built a phylogenetic tree including both the MQ and HQ MAGs and reference genomes belonging to the same order from the GTDB database (see the “Methods” section). In order to generate homogeneously sized datasets, we selected sequences from the 19 order-specific datasets including all the Antarctic MAGs plus all their immediate reference sister taxa (as defined from the corresponding RAxML [51] phylogenetic tree), plus reference representatives of other more distant clades distributed within the tree [37]. The size of the datasets ranged between 46 taxa in the Solibacteriales to 189 taxa for the Corynebacteriales, with most datasets comprising between 50 and 100 taxa. Using a molecular clock approach and available divergence estimates for calibrating the trees [37], we inferred the divergence times of the Antarctic clades from the main tree within each bacterial order. Our phylogenetic and clock analyses indicated that the Antarctic MAGs (red branches in Fig. 2b, c and Supplementary Data) are grouped into ancient monophyletic clades. In some cases, all Antarctic samples form a unique clade within a certain bacterial order, as in Jiangellales, Microtrichales, and UBA5184, while in other cases, we observed a large clustering of Antarctic MAGs interleaved by just one or two reference genomes as in Thermomicrobiales, Solirubrobacteriales, Ktedonobacterales, and Isosphaerales. In almost all other orders (e.g., Acetobacterales, Acidobacteriales, Actinomycetales, Corynebacterales, Frankiales), two or more unrelated Antarctic clades are revealed. Only in a few orders such as Sphingomonadales and Actinomycetales, Antarctic MAGs did not form distinct clades. Our divergence estimates indicate that the vast majority of the Antarctic clades are old (green and orange estimates in Fig. 2a). The diversification of the oldest Antarctic clades occurred on average circa 800 Ma, with estimates ranging from 1.2 billion to 410 Ma (Supplementary Table 6). While the oldest Cyanobacteriales and Ktedonobacterales Antarctic clades are Silurian to Devonian (before 410 Ma), the oldest Antarctic clades in all other orders are pre-Cambrian, with most of them originated in the Tonian (1000-720 Ma).

### Antarctic species encode functions that distinguish them from known references, but are not specific and common to all Antarctic MAGs

**Fig. 3:**
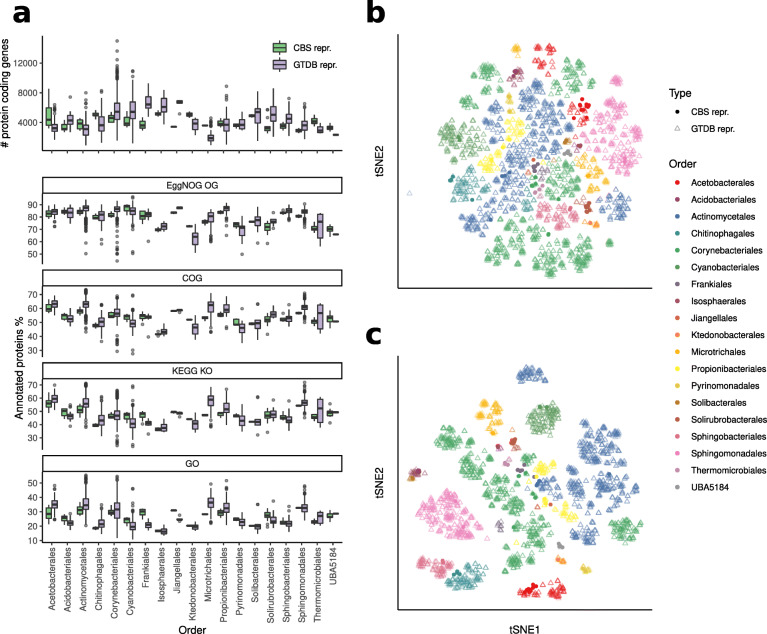
**a** Upper panel: number of protein coding genes in the CBS representatives compared to the reference genomes from the same order; lower panels: fraction of protein coding genes with homologs in EggNOG OGs, COGs, KEGG KOs, and GO. Starting from the Jaccard distances between the protein cluster profiles, **b** or the functional profiles (**c)**, the t-SNE (t-distributed stochastic neighbor embedding) dimensionality reduction highlights the separation between genomes from different orders. Only HQ CBS representative genomes were considered in this analysis

To characterize the set of metabolic functions encoded by the genomes of the Antarctic CBS and identify those that distinguish them from known related species, protein-coding sequences (CDS) have been predicted, clustered together with the CDS of GTDB reference genomes (60% identity, see the “Methods” section), and functionally annotated. We found that, for each CBS, the number of protein-coding genes and the fraction of them with homology to known protein families was usually similar to what was found for GTDB reference genomes of the same order (Fig. 3a, Supplementary Table 8). Moreover, the t-SNE analysis on the 3,836,924 protein clusters showed that the protein profiles are distributed in agreement with the taxonomy at the order level, indicating homogeneous metabolic potential within each order, independently of habitat (Fig. 3b). We could not identify a set of protein clusters that characterize the totality of Antarctic CBS.

We repeated the analysis on proteins for which we could infer a functional annotation from sequence databases. KEGG [52] functional ortholog (KO) profiles were inferred for each genome by EggNOG-mapper and compared to the GTDB representative genomes from the same order. Also in this case, we could not identify a set of functions that characterize the Antarctic CBS across the whole dataset. This is also evident in the t-SNE analysis (Fig. 3c), where Antarctic CBS and reference genomes invariably pooled together according to taxonomy down to the order level, indicating similar functional potential within each order, regardless of provenience, when compared to the functional differences between different orders.

**Fig. 4:**
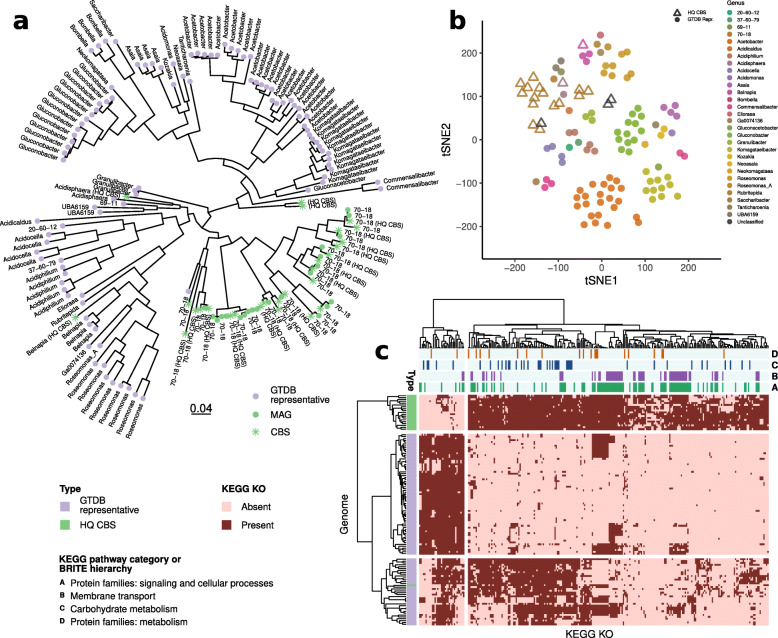
Antarctic CBS form a distinct clade in the order Acetobacterales, family Acetobacteraceae, with characteristic metabolic potential. **a** Maximum-likelihood phylogenetic tree based on GTDB 120 “core” genes including representative genomes (violet) and Antarctic CBS (green). **b** t-SNE (Jaccard distance) based on the protein cluster profiles (60% identity) of the GTDB representatives (circles) and Antarctic high quality CBS (triangles). The representative genomes of Acetobacter aceti e A. aceti_B are not shown. **c** The Fisher’s exact test (Bonferroni corrected *p*<0.01) highlights enriched functional categories. Genomes and KEGG orthologs are clustered according to the Hamming distance between the profiles. The top four KEGG categories significantly more present in the Antarctic CBS are highlighted in the upper bars

However, within each order, the pairwise Jaccard distances between the KO profiles of the Antarctic MAGs were in most cases lower than between MAGs and GTDB representatives (Fig. S9). These data indicated the existence of differences in the functional potential of Antarctic MAGs and taxonomically related reference genomes. In particular, there was evidence that specific functions were overrepresented in distinct phylogenetic clades. For instance, in the Acetobacteraceae family (order Acetobacterales) (Fig. 4a) the t-SNE based on the KO profiles shows a separation of the Antarctic MAGs from the available genomes included in GTDB (Fig. 4b). In particular, the Antarctic clade is enriched (Fisher’s exact test, *p*<0.01, Bonferroni corrected) in genes related to membrane transport, carbohydrate, and amino acid metabolisms by factors from 3 to 31 (Fig. 4c, Table S8). Antarctic Frankiales CBS (Fig. S1a) KO profiles form a group clearly distinct from all known genomes of the same order (Fig. S1 and Supplementary Data). Differently for what observed in the Acetobacterales family, the number of genes (*p*<0.01) belonging to membrane transport, carbohydrate, and amino acid metabolisms that are significantly associated with one clade is higher in the reference genomes by factors of 5, 4, and 9, respectively (Fig. S1d, Supplementary Table 8).

To identify genomic features that might explain its widespread presence, we compared the newly identified Candidatus *Jiangella antarctica *to other species from the genus Jiangella, the only represented in the order Jiangellales (Fig. S2a). We found that Antarctic Jiangellales have the smallest genome sizes, with a significant reduction of the number of genes in several functional categories (Fig. S2b,c and Supplementary Table 9). Moreover, several of the KO that were significantly more represented in the Antarctic genomes were involved in the pathway for carotenoid biosynthesis (Fig. S2d).

Additionally, we compared the eight newly identified CBS from the order Thermomicrobiales (class Chloroflexia) to the known genomes from the same order. We found an increase in genome size, with a number of KEGG pathways, in particular those related to transport, more represented in the newly assembled Antarctic genomes (Fig. S3 and Supplementary Table 10).

## Discussion

Whole-genome metagenomics has contributed substantially to our understanding of global microbial diversity [53]. Here, we retrieved 497 draft MAGs from environmental DNA extracted from eighteen Antarctic cryptoendolithic communities. These newly assembled genomes were clustered into 269 previously uncharacterized species-level groups. Most of these new candidate bacterial species (CBS) could not be taxonomically classified even at higher taxonomic levels; for instance, out of 269, 81 only were assigned to known genera. These findings demonstrated that a large amount of bacterial diversity remains to be genomically characterized across these environments and that the Antarctic endolithic niches represent a reservoir for unknown bacterial taxa.

These MAGs represent the first example of bacterial genomes recovered from these microbial ecosystems; for instance, to date, only a few Cyanobacteria genomes were constructed with more than 93% estimated completeness from Atacama rocky communities [54]. Overall, Antarctic endolithic microbiomes characterized in this study comprised 12 phyla, 22 classes, and 33 orders. The newly assembled MAGs widen the phylogenetic diversity of bacterial tree of life by more than 50% for Jiangellales, Frankiales, Thermomicrobiales, Isosphaerales, Solirubrobacterales, and UBA5184 lineages.

Actinobacteria, Chloroflexi, and Proteobacteria represent the most abundant phyla and the “core” (i.e., present in almost all samples) members of these communities, as previously reported [12, 13]. On the contrary, Deinococci and Cyanobacteria were generally underrepresented. Actinobacteria are not only the main producers of microbial-derived drugs and play an important role associated with plants [55], but they were found to be widely distributed in ecologically different environments, including extreme terrestrial habitats [56, 57] such as hot and cold deserts [58, 59]. Compared with Actinobacteria from temperate habitats, the adaptation strategies of the members of this phylum colonizing extreme environments are still not well understood. Further work is needed to give insights into how this bacterial group adapts to the severe conditions found in desert habitats. Proteobacteria are the dominant component of Polar habitats including soil biotopes [60], cryoconite holes [61], and rock-inhabiting communities in cold climates [61, 62]. Amongst the most representative species, we found one CBS of Jiangellales [63], an order from the class Actinobacteria that encompasses species isolated from different habitats including indoor environments, cold springs on the Qinghai-Tibet Plateau [64], and caves [65, 66]. This CBS, herein named “Candidatus *Jiangella antarctica*,” was present across all samples, suggesting a high adaptation and specialization of this species to the extreme Antarctic environment. The genomes of J. gansuensis, isolated from desert soils in Gansu Province (China) [67], and of Ca. J. antarctica (5.6 and 3.6 Mbp, respectively) showed a smaller size compared with other *Jiangella* species (~ 7 Mbp). Indeed, we found that a significant reduction of the number of genes in several functional categories occurred in the Ca. *J. antarctica*. In particular, genes related to transport, amino, and nucleotide sugar metabolism were underrepresented. These findings support the hypothesis that these microbial species may have undergone a phase of genome reduction to adapt to the hasherst desert conditions to reduce, for instance, the metabolic costs associated with DNA replication and processing. Conversely, genes involved in the carotenoid biosynthesis pathway were enriched in this species, suggesting that the capability to synthesize these pigments is specific to the Antarctic microbes to enhance resistance to UV radiation and freeze-thawing stresses [68, 69].

We found that at least one representative CBS of the class Chloroflexia (i.e., green non-sulfur bacteria), consisting of autotrophic bacteria, was present in all samples. Their capacity of anoxygenic photosynthesis and the presence of bacteriochlorophyll as light-harvesting pigment expand the possibility and the conditions for the community of carbon fixation in highly oligotrophic conditions of the Antarctic desert, a main strategy to conserve energy [70]. Members of Chloroflexi were discovered in Alpine tundra soil, Atacama desert [71] and in microbial mats found in Japanese hot springs [72]. More recently, two novel Chloroflexi, obtained from hot springs in Yellowstone National Park, were identified as putative nitrite-oxidizing bacteria by the presence of nitrite oxidoreductase encoding genes in their genomes [73]. The high abundance of Chloroflexi in such arid environments [74–76] may reflect specific adaptations of this group to survive under arid conditions, but its specific functional role is still to be clarified.

When comparing our newly identified Chloroflexi CBS with the known genomes from the same order, we observed an increase in genome size, with several KEGG pathways, in particular those related to transport, more represented in the newly Antarctic MAGs. This is apparently in contrast to what observed for the genus Jiangella, but it remains rather speculative to generalize considerations at class level.

Our newly assembled MAGs increase by more than 50% the number of representative species in Frankiales (Actinobacteria, G+) that include nitrogen-fixing bacteria in both the free-living and the symbiotic state. Members of *Frankia* genus were found resistant to several stresses such as salinity, heavy metals, extreme pH, and drought [77]. The high recurrence of this group may suggest a critical role in the Antarctic endolithic ecosystems functioning as contributors for nitrogen fixation.

Since a small number of species are shared among all samples analyzed and the majority of CBS were barely detected, we surmise that dispersal may be not the sole determining factor in shaping the diversity and structure of these communities. Dispersal, in these areas, takes place through transportation of microbial propagules associated to rock fragments blown over long distances by the strong winds. Despite the efficiency of this mechanism, a local diversification apparently occurs; very few adapted species can perpetuate in all locations, while biodiversity remains highly variable regardless of geography. Similar conclusions were reached by Archer and colleagues [78], who recently reported that persistent local airborne inputs were unable to fully explain the composition of Antarctic soil communities. Despite the arguably lower sensitivity of shotgun metagenomics compared with amplicon-based methods for biodiversity description [48, 79], our study confirms earlier findings of high site variability between prokaryotic communities in Antarctica soils [80]. The presence of recurrent species in the Antarctic cryptoendolithic communities has been also observed for the fungal counterpart: for instance, the endemic black fungus Friedmanniomyces endolithicus has been reported in almost all samples collected in the Victoria Land in more than 20 years of Antarctic Campaigns [81, 82], indicating a high degree of adaptation to the prohibitive environmental conditions of this area.

Our molecular clock analysis indicated that most of the Antarctic bacterial clades found here originated during the Tonian glaciations, in a period ranging from 800 to 1000 Ma. before the many glaciations of the Cryogenian [83, 84] when Antarctica was still part of the Supercontinent Rodinia. Even accounting for the uncertainties of the estimated divergence times (see bars in Fig. 2) and the many prior assumptions embedded in the molecular clock of Antarctic organisms [85], our data exclude the hypothesis that the evolution of these bacterial clades was driven in response to the environmental pressure of the more recent Antarctica geological history. In fact, the last cooling events started once Antarctica reached the South Pole in the early Oligocene (~34 Ma), after the separation from Gondwanaland about 200 Ma [86], while the present icy conditions were established round 3 Ma. Our results suggest that these new bacterial clades diversified from a pool of pre-existing frost-evolved species that found the opportunity to spread in Antarctica once the present conditions were established. Based on these data we cannot establish when these organisms reached the continent, but it could be expected that such old clades, or their relatives, may be found searching elsewhere in extreme-cold niches, possibly in continents that were neighboring Antarctica in the era of the Supercontinent Rodinia (i.e., North America, India or Australia). This accomplishes the scenario of “everything is everywhere, but the environment selects” suggested in 1934 by Baas Becking [87]. Similar results were found in a global survey of the hypolithic cyanobacterial genus *Chroococcidiopsis*, where a molecular phylogenetic analysis found that variants from hot and cold deserts were grouped in different lineages, with an estimated time to last common ancestor of the hot and cold clade of ~2400 Ma and regional genetic variability maintained over geological timescales [88].

Further, whole-genome metagenomic sequencing can be employed to investigate not only the composition of the microbial communities, but also the functional roles that these community members may play. In our study, we showed that the set of Antarctic MAGs predicted proteins, typically part of primary metabolism playing a role in normal growth and survival, was significantly consistent with existing representatives in the public domain from the same order. Whereas, at the order level, de novo protein clustering and functional annotation confirmed the results of the phylogenetic analysis indicating that several CBS form separate lineages. The main functional processes which appeared to be potentially enriched in the Acetobacteraceae compared with reference genomes were those related to amino acid and carbohydrate metabolisms, containing proteins with high identity with similar protein sequences in the public domain, while these pathway categories were underrepresented in the order Frankiales. The functional differences observed may be related to a specific adaptation to the Antarctic endolithic niche.

The release of the endolithic MAGs presented here will surely remodel the way we interpret and explore the Antarctic ecosystems data. A more detailed examination of such genomes and additional samples will further increase our understanding of microbial evolution and metabolic diversity and provide important insights into the role of these microorganisms in Antarctic desert functioning.

## Conclusions

In conclusion, our data report for the first time the genomes of the dominant bacterial species in Antarctic cryptoendolithic communities; none of the 269 CBS individuated were accounted to already described taxa. Most of the new species found are organized into ancient monophyletic clades that differentiated from known bacterial orders in a time range that predates the estimated origin of modern Antarctica and the establishment of the present glacial climate. Our data point toward a scenario where extant Antarctic bacterial clades are the remnants of ancient bacterial lineages, dating back up to 1000 Ma, which found in the present frost conditions of the continent a new opportunity to spread and diversify. These findings give also new insights for the possibility of life beyond the Earth (e.g., on Mars) since microbial life, if ever evolved, may have escaped extinction for a timescale of evolutionary significance in proper refugia. 

Despite the variability of the bacterial assemblages observed among samples, a “core” of few species was shared among all specimens examined. We did not find a specific set of functions that characterize the Antarctic MAGs; yet, genes for several metabolic pathways were differently represented (both over- or underrepresented, depending on the group considered) compared to reference genomes. A deeper understanding of these mechanisms is likely to contribute substantially to our capacity to predict how these ecosystems respond to the projected climate change which is particularly enhanced at the Poles. Moreover, it would be possible to extrapolate this information in worldwide arid areas deepening our comprehension of the service that these communities provide in an era of rapid desertification.

## Supplementary Information

**Additional file 1: Figure S1.** Antarctic CBS form two distinct clades in the order Frankiales, with characteristic metabolic potential. **a)** Maximum-likelihood phylogenetic tree based on GTDB 120 core genes including representative genomes (violet) and Antarctic CBS (green). **b)** Principal Coordinate Analysis (Jaccard distance) of the protein cluster profiles (60% identity) **b)** and of the metabolic potential. **d)** The Fisher’s exact test (Bonferroni corrected *p*<0.01) highlights enriched functional categories. Genomes and KEGG orthologs are clustered according to the Hamming distance between the profiles. The top four KEGG categories significantly more present in the Antarctic CBS are highlighted in the upper bars. **Figure S2.** Antarctic *Jiangellales* CBS reveal a substantial genome reduction compared to known species, with characteristic differences in metabolic potential. **a)** Maximum-likelihood phylogenetic tree based on GTDB genes, including representative genomes from the GTDB database (violet) and Antarctic CBS (green). **b)** KEGG orthologs that are significantly less frequent in Antarctic Jiagellales compared to reference (uncorrected *p*<0.05, Fisher’s exact test). Only the first 25 pathways (ranked by the total number of significant orthologs) are shown. **c)** Number of predicted protein coding sequences in Antarctic (green) and reference (violet) Jiangellales **d)** The heatmap shows the presence (dark green) of KEGG orthologs belonging to the carotenoid biosynthesis pathway. The only gene involved in carotenoid biosynthesis detected in both CBS and GTDB reference genomes is the crtD. **e)** The phylogenetic tree inferred on the crtD gene highlights a segregation of Antarctic *Jiangellales*. **Figure S3.** Antarctic *Thermomicrobiales* ( class *Chloroflexia* ) CBS reveal characteristic metabolic potential. **a)** The Fisher’s exact test (uncorrected *p*<0.05) highlights a significant presence, in Antarctic genomes, of orthologs involved in transport, compared to the reference *Thermomicrobiales* genomes. Only the first 30 pathways (ranked by the total number of orthologs called significant) are shown. **b)** The prediction of protein coding sequences shows an increment of the number of genes in Antarctic *Thermomicrobiales* compared to reference genomes. **Figure S4.** Distribution of the number of CBS that are specific to a given number of samples, taxonomically classified at the Class level. We identified a set of 10 CBS (belonging to the classes Actinobacteria and Alphaproteobacteria) that are present in at least 75% (14/18) of the samples. **Figure S5.** Mash Screen was used to validate the presence of CBS in the Antarctic samples. **a)** Distribution of the number of CBS marked as present by the containment score estimated by Mash screen. 1009 out of 1094 (92.2%) CBS have been confirmed by Mash (containment score >0.95, green dashed vertical line). **b)** Distribution of the number of CBS marked as present by the estimated multiplicity. **Figure S6.** Scatter plot of the ANI estimated by mapping versus the containment scores estimated by Mash screen for each sample. Horizontal and vertical dashed lines represent the ideal species-level threshold of 0.95 for the containment score and the estimated ANI, respectively. **Figure S7. a)** Percentage of reads that could be mapped to the CBS representatives, grouped by Class. **b)** Per sample percentage of the reads that could be mapped to the CBS representatives, grouped by Class. **Figure S8.** The “ *Candidatus Jiangella antarctica* ” was found in each sample. **a)** Scatter plot of the ANI estimated by mapping versus the containment scores estimated by Mash screen (*p* < 1.47x10 ^-21^ ). **b)** Scatter plot of the median depth of coverage estimated by mapping versus the median multeplicity estimated by Mash. The line of equality is represented in black. **Supplementary Figure S9.** Jaccard distance between the KEGG functional profiles for each Order.

**Additional file 2: Supplementary Table 1.** Results of the CBS detection procedure and the validation using Mash Screen. Each row reports: CBS ID (i.e. the CBS MAG representative), metagenomic sample, estimated depth of coverage (mean, standard deviation, first quartile, median third quartile), number of mapped reads, ANI between the consensus sequences and the CBS representative, coverage breadths at depths from 1 to 5, Mash Screen containment score, number of shared hashes, median multiplicity and containment score p-value. **Supplementary Table 2.** Assembly statistics and taxonomic classification of the MAGs. **Supplementary Table 3.** Abundance of CBS at phylum level, expressed as percentage of reads that could be mapped to the representative CBS. Median: median; Q1 and Q3: first and third quartile; IQR: interquartile range; Mean: mean; SD: standard deviation; #CBS: number of candidate bacterial species belonging to the phylum. **Supplementary Table 4.** Increase in the number of bacterial species for each taxonomic Order provided by the data in the present study, compared to the data available in the GTDB database. **Supplementary Table 5.** Sample metadata. Geographic coordinates of the sampling sites, accession numbers of the raw sequences, accession numbers and N50 of the assembled metagenomes on the JGI IMG/M portal. **Supplementary Table 6.** Prevalence and taxonomic classification for each CBS representative. **Supplementary Table 7.** Summary of Bayesian divergence estimates. For each order we report the mean age of its origin (OO: the split of the order from the closest order) and the 95% CI (OO max and OO min), the origin of the oldest uniquely Antarctic clade (AOO1, the split of the Antarctic clade from a non-Antarctic lineage of the same order), and, where present, the origin of the second oldest antarctic clade (AOO2). See Supplementary Data 1. **Supplementary Table 8.** Number of predicted proteins (NProts) and of proteins that had a match in the EggNOG database (NHitsOG) and that could be associated to a term in the Gene Ontology (NHitsGO) or had a match in the KEGG and COG databases (NHitsKEGG and NHitsCOG, respectively). **Supplementary Table 9.** Number of KEGG orthologs characteristic of the Antarctic or reference *Jiangellales* genomes. The Fisher’s exact test (uncorrected *p*<0.05) was performed to identify unevenly distributed orthologs between the two groups. **Supplementary Table 10.** Number of KEGG orthologs characteristic of the Antarctic or reference *Thermomicrobiales* genomes. The Fisher’s exact test (uncorrected *p*<0.05) was performed to identify unevenly distributed orthologs between the two groups.

**Additional file 3: Supplementary data.**

## Data Availability

Raw metagenomes reads and assemblies are deposited under the NCBI accession numbers listed in Supplementary Table 5. Metagenome assemblies, gene predictions, and JGI annotations are available in the IMG/M web site (https://img.jgi.doe.gov) and in the zenodo repository (https://zenodo.org/record/3610489; DOI: 10.5281/zenodo.3610489). MAGs, translated coding sequences and annotations for high-quality MAGs, metadata, and Candidatus *Jiangella antarctica* ribosomal rRNA genes are available at the zenodo repository (DOI: 10.5281/zenodo.3671352).
