# Supplementary material for: Pre-Cambrian roots of novel Antarctic cryptoendolithic bacterial lineages
Source: Microbiome. 2021 Mar 19;9:63. doi: 10.1186/s40168-021-01021-0 (PMC7980648; doi:10.1186/s40168-021-01021-0)
Supplement: Supplementary file 2 — Additional file 1: Figure S1. Antarctic CBS form two distinct clades in the order Frankiales, with characteristic metabolic potential. a) Maximum-likelihood phylogenetic tree based on GTDB 120 core genes including representative genomes (violet) and Antarctic CBS (green). b) Principal Coordinate Analysis (Jaccard distance) of the protein cluster profiles (60% identity) b) and of the metabolic potential. d) The Fisher’s exact test (Bonferroni corrected p<0.01) highlights enriched functional categories. Genomes and KEGG orthologs are clustered according to the Hamming distance between the profiles. The top four KEGG categories significantly more present in the Antarctic CBS are highlighted in the upper bars. Figure S2. Antarctic Jiangellales CBS reveal a substantial genome reduction compared to known species, with characteristic differences in metabolic potential. a) Maximum-likelihood phylogenetic tree based on GTDB genes, including representative genomes from the GTDB database (violet) and Antarctic CBS (green). b) KEGG orthologs that are significantly less frequent in Antarctic Jiagellales compared to reference (uncorrected p<0.05, Fisher’s exact test). Only the first 25 pathways (ranked by the total number of significant orthologs) are shown. c) Number of predicted protein coding sequences in Antarctic (green) and reference (violet) Jiangellales d) The heatmap shows the presence (dark green) of KEGG orthologs belonging to the carotenoid biosynthesis pathway. The only gene involved in carotenoid biosynthesis detected in both CBS and GTDB reference genomes is the crtD. e) The phylogenetic tree inferred on the crtD gene highlights a segregation of Antarctic Jiangellales. Figure S3. Antarctic Thermomicrobiales ( class Chloroflexia ) CBS reveal characteristic metabolic potential. a) The Fisher’s exact test (uncorrected p<0.05) highlights a significant presence, in Antarctic genomes, of orthologs involved in transport, compared to the reference Thermomicrobiales genomes [file 40168_2021_1021_MOESM1_ESM.pdf]

Supplementary Figures

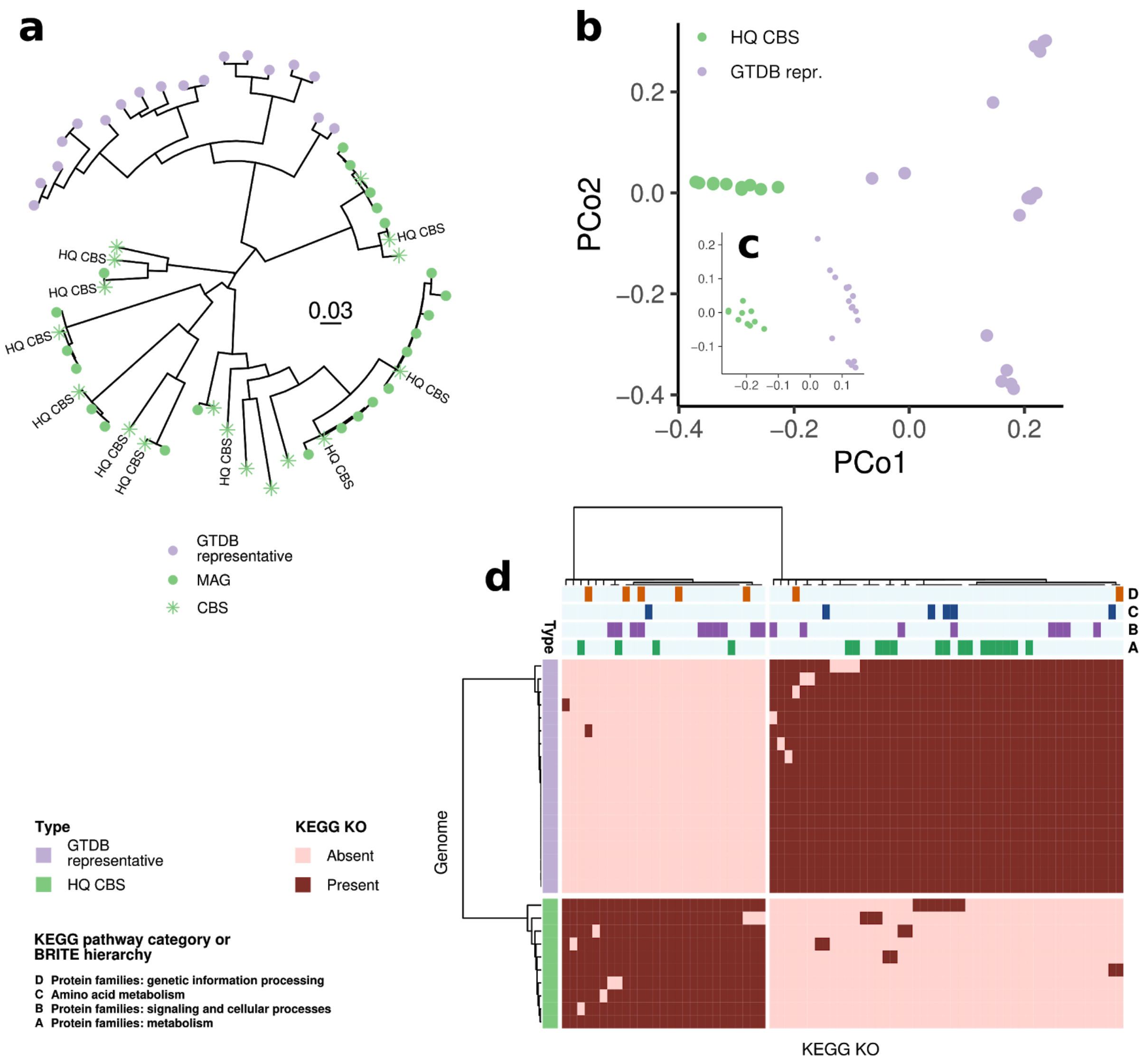

Figure S1. Antarctic CBS form two distinct clades in the order Frankiales, with characteristic metabolic potential. **a)** Maximum-likelihood phylogenetic tree based on GTDB 120 core genes including representative genomes (violet) and Antarctic CBS (green). **b)** Principal Coordinate Analysis (Jaccard distance) of the protein cluster profiles (60% identity) **b)** and of the metabolic potential. **d)** The Fisher's exact test (Bonferroni corrected $p<0.01$) highlights enriched functional categories. Genomes and KEGG orthologs are clustered according to the Hamming distance between the profiles. The top four KEGG categories significantly more present in the Antarctic CBS are highlighted in the upper bars.

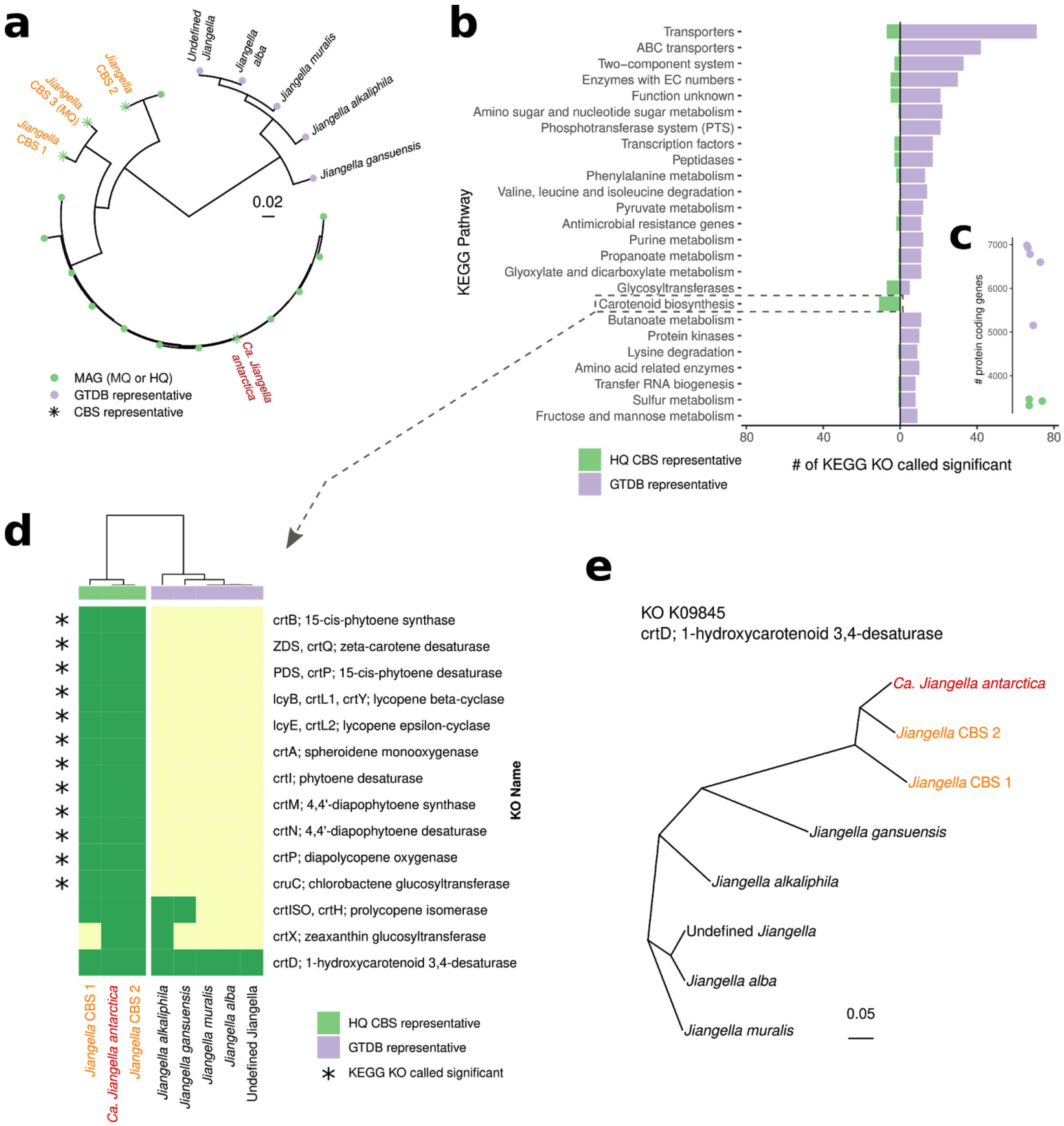

Figure S2. Antarctic *Jiangellales* CBS reveal a substantial genome reduction compared to known species, with characteristic differences in metabolic potential. **a)** Maximum-likelihood phylogenetic tree based on GTDB genes, including representative genomes from the GTDB database (violet) and Antarctic CBS (green). **b)** KEGG orthologs that are significantly less frequent in Antarctic Jiagellales compared to reference (uncorrected $p<0.05$, Fisher's exact test). Only the first 25 pathways (ranked by the total number of significant orthologs) are shown. **c)** Number of predicted protein coding sequences in Antarctic (green) and reference (violet) Jiangellales **d)** The heatmap shows the presence (dark green) of KEGG orthologs belonging to the carotenoid biosynthesis pathway. The only gene involved in carotenoid biosynthesis detected in both CBS and GTDB reference genomes is the crtD. **e)** The phylogenetic tree inferred on the crtD gene highlights a segregation of Antarctic *Jiangellales***.**

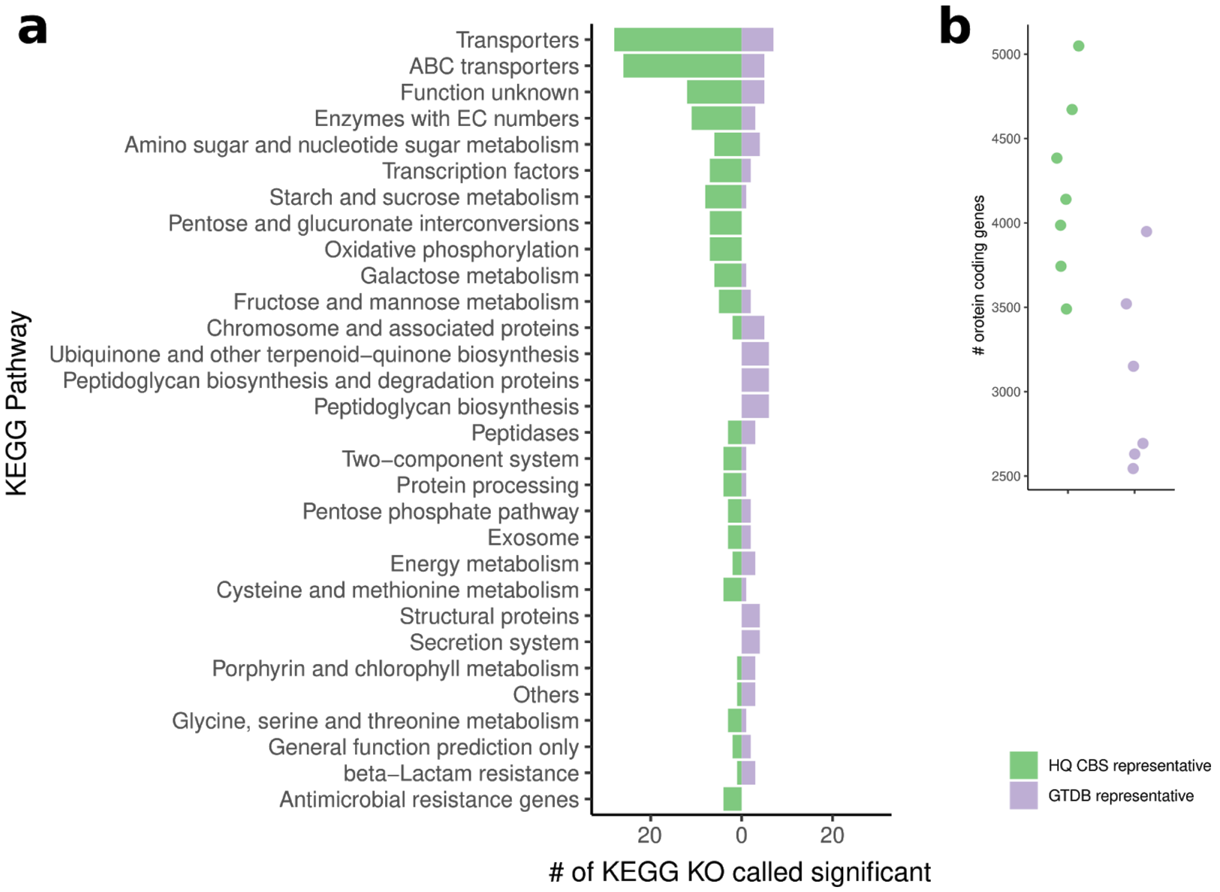

Figure S3. Antarctic *Thermomicrobiales* (class *Chloroflexia*) CBS reveal characteristic metabolic potential. **a)** The Fisher's exact test (uncorrected p<0.05) highlights a significant presence, in Antarctic genomes, of orthologs involved in transport, compared to the reference *Thermomicrobiales* genomes. Only the first 30 pathways (ranked by the total number of orthologs called significant) are shown. **b)** The prediction of protein coding sequences shows an increment of the number of genes in Antarctic *Thermomicrobiales* compared to reference genomes.

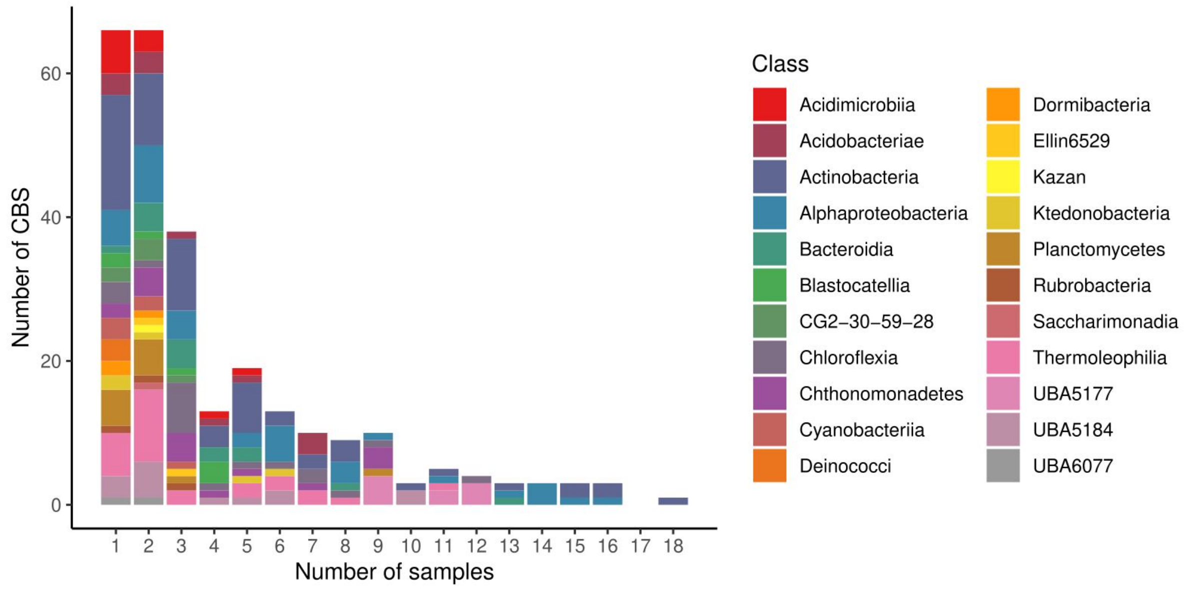

Figure S4. Distribution of the number of CBS that are specific to a given number of samples, taxonomically classified at the Class level. We identified a set of 10 CBS (belonging to the classes Actinobacteria and Alphaproteobacteria) that are present in at least 75% (14/18) of the samples.

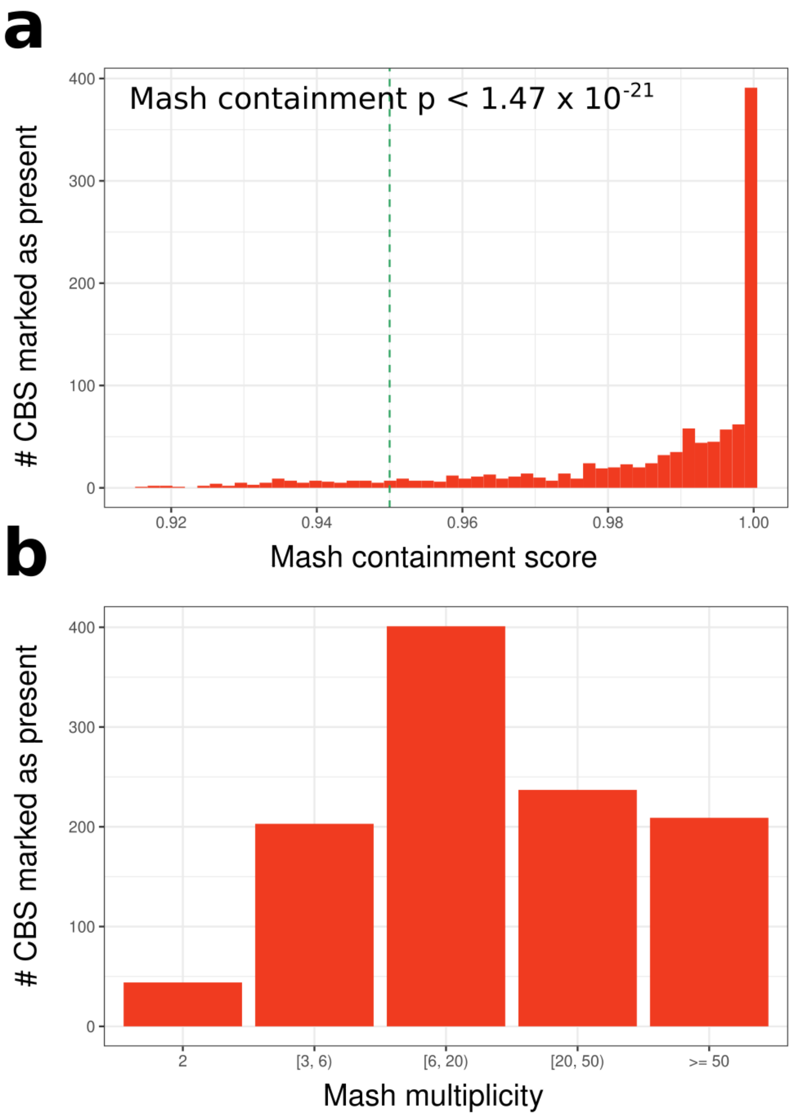

Figure S5. Mash Screen was used to validate the presence of CBS in the Antarctic samples. **a)** Distribution of the number of CBS marked as present by the containment score estimated by Mash screen. 1009 out of 1094 (92.2%) CBS have been confirmed by Mash (containment score >0.95, green dashed vertical line). **b)** Distribution of the number of CBS marked as present by the estimated multiplicity.

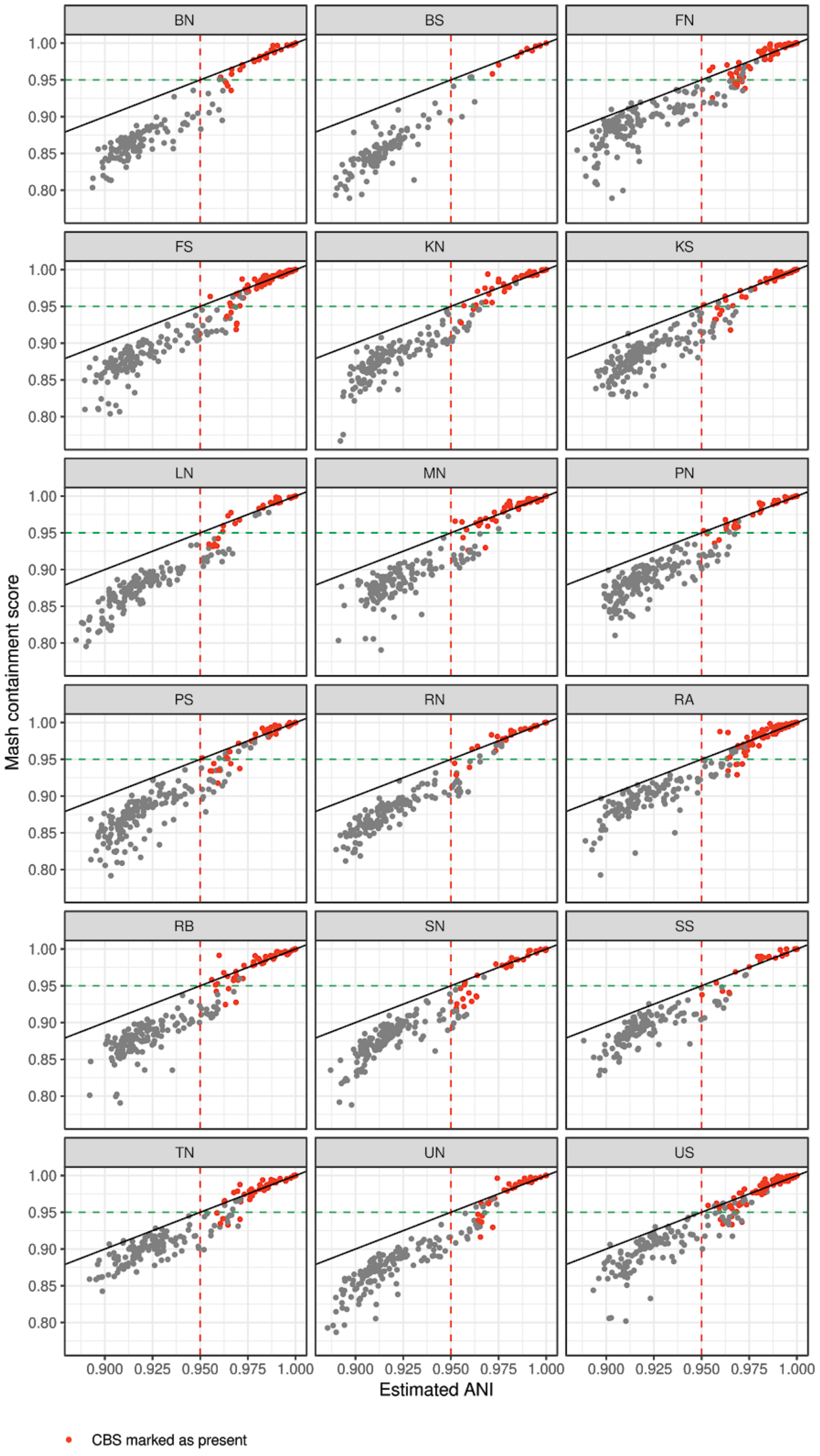

Figure S6. Scatter plot of the ANI estimated by mapping versus the containment scores estimated by Mash screen for each sample. Horizontal and vertical dashed lines represent the ideal species-level threshold of 0.95 for the containment score and the estimated ANI, respectively.

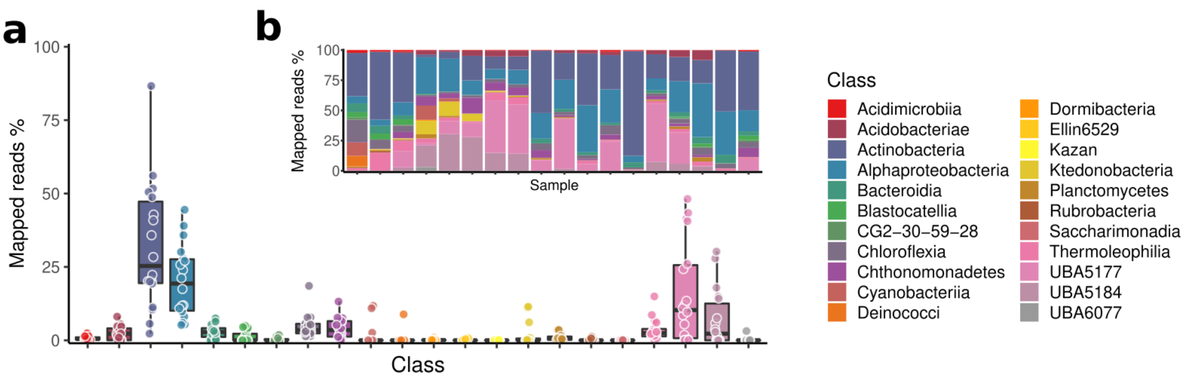

Figure S7. a) Percentage of reads that could be mapped to the CBS representatives, grouped by Class. **b)** Per sample percentage of the reads that could be mapped to the CBS representatives, grouped by Class.

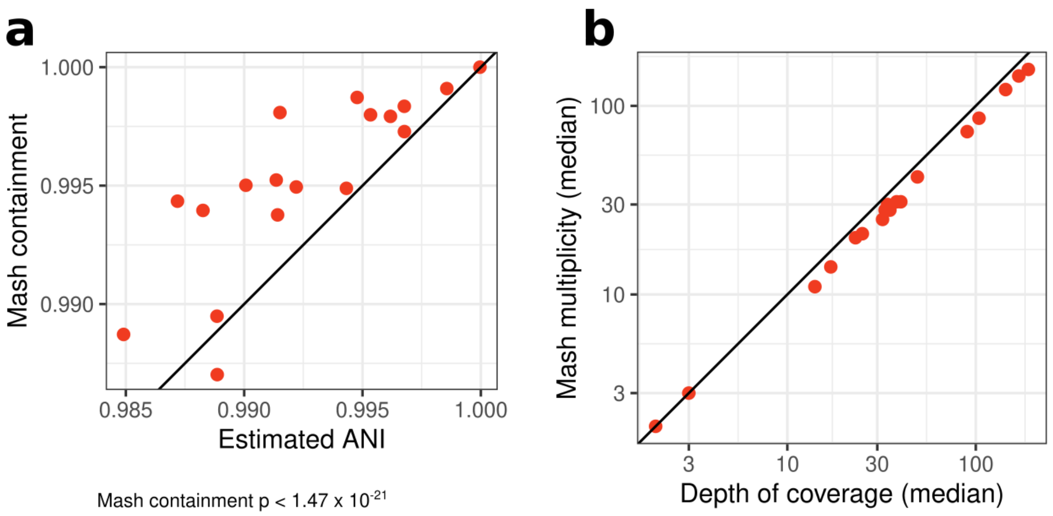

Figure S8. The "*Candidatus Jiangella antarctica*" was found in each sample. **a)** Scatter plot of the ANI estimated by mapping versus the containment scores estimated by Mash screen ($p < 1.47 \times 10^{-21}$). **b)** Scatter plot of the median depth of coverage estimated by mapping versus the median multeplicity estimated by Mash. The line of equality is represented in black.

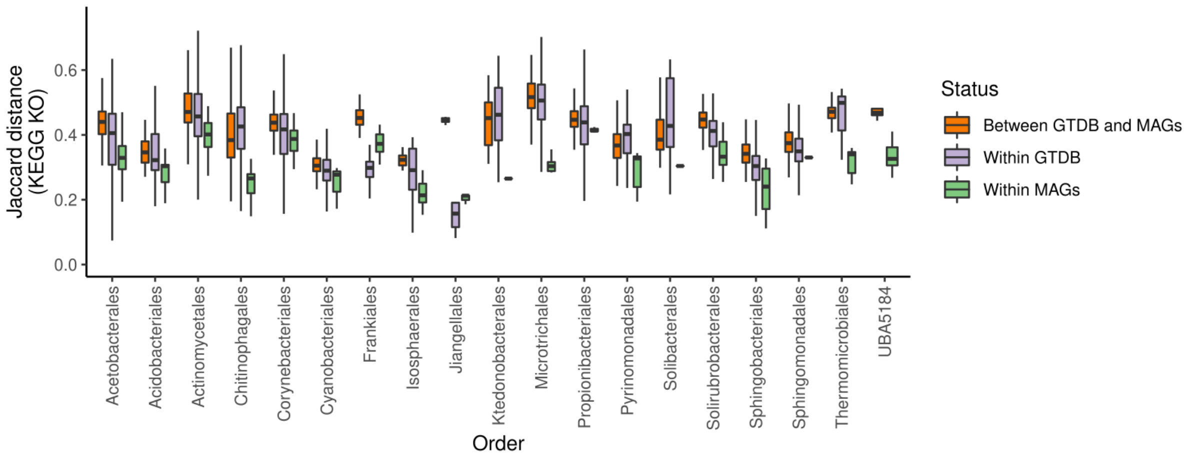

Supplementary Figure S9. Jaccard distance between the KEGG functional profiles for each Order.

Supplementary Table Captions

Supplementary Table 1. Results of the CBS detection procedure and the validation using Mash Screen. Each row reports: CBS ID (i.e. the CBS MAG representative), metagenomic sample, estimated depth of coverage (mean, standard deviation, first quartile, median third quartile), number of mapped reads, ANI between the consensus sequences and the CBS representative, coverage breadths at depths from 1 to 5, Mash Screen containment score, number of shared hashes, median multiplicity and containment score p-value.

Supplementary Table 2. Assembly statistics and taxonomic classification of the MAGs.

Supplementary Table 3. Abundance of CBS at phylum level, expressed as percentage of reads that could be mapped to the representative CBS. Median: median; Q1 and Q3: first and third quartile; IQR: interquartile range; Mean: mean; SD: standard deviation; #CBS: number of candidate bacterial species belonging to the phylum.

Supplementary Table 4. Increase in the number of bacterial species for each taxonomic Order provided by the data in the present study, compared to the data available in the GTDB database.

Supplementary Table 5. Sample metadata. Geographic coordinates of the sampling sites, accession numbers of the raw sequences, accession numbers and N50 of the assembled metagenomes on the JGI IMG/M portal.

Supplementary Table 6. Prevalence and taxonomic classification for each CBS representative.

Supplementary Table 7. Summary of Bayesian divergence estimates. For each order we report the mean age of its origin (OO: the split of the order from the closest order) and the 95% CI (OO max and OO min), the origin of the oldest uniquely Antarctic clade (AOO1, the split of the Antarctic clade from a non-Antarctic lineage of the same order), and, where present, the origin of the second oldest antarctic clade (AOO2). See Supplementary Data 1.

Supplementary Table 8. Number of predicted proteins (NProts) and of proteins that had a match in the EggNOG database (NHitsOG) and that could be associated to a term in the Gene Ontology (NHitsGO) or had a match in the KEGG and COG databases (NHitsKEGG and NHitsCOG, respectively).

Supplementary Table 9. Number of KEGG orthologs characteristic of the Antarctic or reference *Jiangellales* genomes. The Fisher's exact test (uncorrected $p<0.05$) was performed to identify unevenly distributed orthologs between the two groups.

Supplementary Table 10. Number of KEGG orthologs characteristic of the Antarctic or reference *Thermomicrobiales* genomes. The Fisher's exact test (uncorrected $p<0.05$) was performed to identify unevenly distributed orthologs between the two groups.
